# Accumulation of intestinal tissue 3-deoxyglucosone attenuated GLP-1 secretion and its insulinotropic effect in rats

**DOI:** 10.1186/s13098-016-0194-9

**Published:** 2016-11-29

**Authors:** Lurong Zhang, Xiudao Song, Liang Zhou, Guoqiang Liang, Heng Xu, Fei Wang, Fei Huang, Guorong Jiang

**Affiliations:** Suzhou Academy of Wumen Chinese Medicine, Suzhou Hospital of Traditional Chinese Medicine, No. 18, Yangsu Road, Suzhou, 215003 Jiangsu People’s Republic of China

**Keywords:** 3-Deoxyglucosone, Glucagon-like peptide-1, Insulinotropic, Impaired glucose regulation, Type 2 diabetes mellitus

## Abstract

**Background:**

Our recent findings support the idea that 3-deoxyglucosone (3DG), a dietary composition, has been suggested as an independent factor for the development of prediabetes. Secretion of glucagon-like peptide-1 (GLP-1) has been suggested to be impaired in T2DM and in conditions associated with hyperglycemia. Since low oral bioavailability of 3DG has been indicated in a single administration study, in the present study we examined if 3DG is capable of accumulating in intestinal tissue of rats after 2-week administration of 3DG, and the 3DG treatment affects GLP-1 secretion and glucose tolerance.

**Methods:**

Rats were administered by gastric gavage for 2 weeks. We measured 3DG contents of intestinal tissues (by HPLC), plasma levels of total GLP-1 (by ELISA), insulin and glucagon (both by radioimmunoassay) and blood glucose concentrations. The expressions of the sweet receptor subunits (TAS1R2, TAS1R3) and its downstream molecule TRPM5 in duodenum and colon tissues of rats were quantified by WB. We examined GLP-1 secretion in enteroendocrine STC-1 cells exposured to 3DG.

**Results:**

3DG treatment for 2 weeks increased 3DG content of intestinal tissues, fasting blood glucose concentration, and reduced plasma concentrations of GLP-1 and insulin at fasting and 15 and 180 min after the glucose load and oral glucose tolerance in conjunction with increased plasma glucagon concentrations. The expressions of TAS1R2, TAS1R3 and TRPM5 were shown to be reduced whereas 3DG treatment did not affect plasma dipeptidyl peptidase-4 activity, indicating an impaired GLP-1 secretion in 3DG-treated rats. This idea was further supported by the fact that exposure to 3DG directly decrease GLP-1 secretion in STC-1.

**Conclusion:**

It is the first demonstration that 3DG was capable of accumulating in intestinal tissue and thereby decreased secretion of GLP-1 and insulin in a similar manner. 3DG-treated rats developed impaired glucose regulation (IGR) with obviously pancreatic islet cell dysfunction. It is further concluded that a decrease in the biological function of GLP-1 resulting from the decreased GLP-1 secretion is the most likely mechanism for the impaired insulin secretion, which ultimately promoted the development of IGR. These results will also contribute to a better understanding of the significance for restoring physiological GLP-1 secretion.

**Electronic supplementary material:**

The online version of this article (doi:10.1186/s13098-016-0194-9) contains supplementary material, which is available to authorized users.

## Background

Dietary changes in overall structure have been clearly shown to be associated with the development of diabetes [1]. Aside from obviously increased intake of caloric and amounts of dietary fat, both of which have been demonstrated to be important to the development of prediabetes [2], the changes also toward increased sweetening of the diet, food additives intake, by-products during food processing or storing and other important elements [3]. Artificial sweeteners (e.g., saccharin) have been found to implicate in the development of obesity and obesity-related metabolic syndrome, associated with the alterations in composition and function of the intestinal microbiota [4]. It is worth noting that 1,2-dicarbonyl compounds [5–7] and advanced glycation end products (AGEs) [8, 9], both of which are easily formed from carbohydrates in caramelization course and Maillard reactions in food, have been reported to increase the risk of type 2 diabetes mellitus (T2DM) and its complications. Based on an investigation of the content of 1,2-dicarbonyl compound in a great variety of commonly consumed foods, 3-deoxyglucosone (3DG) was proved to be the predominant 1,2-dicarbonyl compound [10]. In addition to intensively investigate as a precursor for AGEs, 3DG itself has certain biological activities [11–13], specifically on the ability to induce insulin resistance in vitro [13]. The further reports in clinical and animal research indicated that 3DG had been linked to an impaired glucose tolerance [6, 14, 15], thereby constituting an independent factor for the development of prediabetes.

The term “enteroinsular axis” refers to the signaling pathways between the gut and pancreatic islets that regulate blood glucose homeostasis [16]. The pathogenesis of T2DM is associated with a defect in this enteroinsular axis [17, 18]. The signaling pathways in gut related to regulation of glucose homeostasis are mediated by gut hormone, microbiota or immune system and those have gradually been a therapeutic target for diabetes. Glucagon-like peptide-1 (GLP-1) is an important gut hormone that can act via the enteroinsular axis to potentiate insulin secretion from pancreatic islets β-cell, known as the incretin effect [19]. Owing to the incretin effect, analogs of GLP-1, GLP-1 receptor agonists and dipeptidyl peptidase-IV (DPP-IV) inhibitors are available as treatments for T2DM [20]. GLP-1 secretagogues also represent a potential approach to enhance incretin action in T2DM. Actually, increasing endogenous GLP-1 secretion by dietary non-digestible ingredient (e.g., resistant maltodextrin and oligofructose), has been shown to improve glucose tolerance [21, 22]. Reduced plasma GLP-1 concentrations were sometimes observed in T2DM [23–25] even prediabetes [26] stages, which may provide an explanation to the markedly impaired incretin effect in patients with T2DM [27] in addition to the deficient in the β-cell response to GLP-1 after meal ingestion [20]. Impairment of GLP-1 action caused by a blunted secretion of L-cells was also observed in early states of T2DM [28]. Impairment of GLP-1 secretion, therefore, has been also proposed to be associated with a reduced glucose-stimulated insulin secretion and an impaired glucose tolerance [29].

Considering the significance of the incretin effect of GLP-1, the factors related to harmful effects towards endogenous GLP-1 secretion become very important. To our knowledge, some endogenous or exogenous events that may decrease GLP-1 secretion have been investigated involving of the direct regulation of GLP-1-secreting cell, but the studies tend to be few. Stimulated hyperlipidemia and a high fat diet given to mice induce a reduction of the number of GLP-1-secreting cells in vitro and in vivo [30]. In one more in vitro study, lipopolysaccharide, a gut bacterial product, was found to induce the apoptosis in intestinal endocrine cell line STC-1 in a dose-dependent manner [31]. Thus, continuing to seek other factors that potentially harm GLP-1 secretion would help to restore physiological GLP-1 secretion and deserve to be explored. In an earlier study, 3DG was absorbed into the systemic circulation at a percentage of about 1‰ 2 h after single oral administration of 3DG [32], suggesting the absorption rate of 3DG from foodstuffs is very slow. This result raises the possibility that 3DG has the ability to affect GLP-1 secretion. We therefore investigated if 3DG is capable of accumulating in intestinal tissue where it may have a role in GLP-1 axis after continuous oral administration of 3DG.

In the current study, 3DG was administered by gastric gavage to Sprague–Dawley (SD) rats for 2 weeks to investigate the distribution of 3DG in intestinal tissues. We also examined the effects of intragastric administration of 3DG on plasma levels of GLP-1, insulin and glucagon, and glucose regulation. Furthermore, the expressions of the sweet receptor subunits (TAS1R2, TAS1R3) and its downstream molecule TRPM5 in duodenum and colon tissues of rats, which is related to GLP-1 secretion, were investigated. In addition, we used the STC-1 L-cell model to investigate the direct effect of 3DG on GLP-1 secretion.

## Methods

### Synthesis of 3DG

According to the method of Kato et al. [32], 3DG was synthesized from glucose as previously described [13].

### Determination of appropriate doses of intragastric administration of 3DG

Previous reports have estimated an average dietary 3DG intake of about 50 mg/day based on the 3DG content in commonly consumed foods [10]. In order to achieve the equivocal effect of a potential 3DG intake of 50 mg per day, we calculated a dose based on body surface area (4.5 mg/kg for rats). Previously, we have reported that intragastric administration of 5 mg/kg 3DG for 2 weeks lightly increased plasma glucose level under oral glucose tolerance tests in mice. Therefore, we gave 5, 20 or 50 mg/kg 3DG by gastric gavage.

### Animals

11-week-old SD rats were purchased from Matt Albert Technology Co. Ltd (Suzhou, China) and housed in a temperature-controlled room (23 °C) and 12 h light/12 h dark cycle. All of animal experimental procedures were conducted in compliance with Guide for care and use of laboratory animals (Eighth edition, 2011). The study was approved by the local ethic committee of Suzhou Hospital of Traditional Chinese Medicine. The rats had free access to a standard rodent chow diet (Shuangshi Laboratory Animal Feed Science Co. Ltd, Suzhou, China) and water. The diet contained water (≤10%), crude proteins (≥20.5%), crude fat (≥4%), crude fiber (≤5%), crude ash (≤8%) and mixture of vitamins and micronutrients. After 1 week of acclimatization, the rats were randomly divided into four groups with similar fasting glucose concentration, and each group consisted of six rats. Vehicle (control), 5 mg/kg 3DG, 20 and 50 mg/kg 3DG were given by gastric gavage daily with an administrated period of 2 weeks. Body weight was measured daily. The rats were fasted overnight before the experiments.

### STC-1 cells culture

STC-1 cells, an enteroendocrine intestinal cell line, were obtained from Cell Bank of the Chinese Academy of Sciences (Shanghai, China). The cells were grown in Dulbecco’s modified Eagle’s medium (DMEM; Gibco; Thermo Fisher Scientific, Inc., Waltham, MA, USA) containing 15% (v/v) horse serum, 2.5% (v/v) fetal bovine serum (FBS; Zhejiang Tianhang Biological Technology Co., Ltd., Huzhou, China), and 25 mmol/L glucose at 37 °C in a 5% CO_2_ humidified atmosphere. The cells were grown to 70–80% confluence for the experiments.

### Oral glucose tolerance test (OGTT)

After fasting overnight, a basal blood sample was collected from a tail vein for the measurement of fasting glucose levels using a glucose meter (ACCU-CHEK, Roche, US). Then, the rats were fed with glucose by gastric gavage (2.5 g/kg). And additional blood samples were collected from tail vein at 0, 30, 60, 90, 120 and 180 min following the glucose load, and glucose concentration was determined with a glucose meter. The area under the glycaemic curves (AUC) were calculated for each group of rats.

### Measurements of GLP-1, GIP, insulin and glucagon in plasma

Blood samples from aorta abdominalis were collected at 15 and 180 min points following the glucose load for the measurements of insulin, glucagon, GLP-1 (total). Plasma levels of insulin and glucagon were assayed with the corresponding radioimmunoassay kits (Beijing North Institute of Biological Technology, Beijing, China). Plasma GLP-1 concentration was measured using the ELISA kits (Millipore, MA, USA). Total GLP-1 includes both intact [GLP-1-(7–36) amide and GLP-1-(7–37)] and inactivated forms of GLP-1 (GLP-19–36 amide and GLP-1 9–37 degraded by DPP-4).

### Measurement of plasma dipeptidyl peptidase-4 (DPP-4) activity

According to the method of Pederson et al. [33], plasma DPP-4 activity was determined by a colorimetric assay, using H-Gly-Pro-*p*-nitroanilide (Sigma, St Louis, MO, USA) as a substrate.

### Distribution of 3DG in intestinal tissues after treatment with exogenous 3DG

After 2 weeks of intragastric administration of 3DG, the rats were then killed and intestinal tissues were collected for the measurement of 3DG contents by HPLC. Before the measurement, the content of gastrointestinal tract was completely removed.

### Western blot analysis

In rats treated with 50 mg/kg 3DG, the duodenum and colon tissues were collected 2 weeks after intragastric administration 3DG. Methods for quantification of whole protein content and western blot have been described previously [13]. Antibodies against TAS1R2, TAS1R3 and TRPM5 were obtained from Cell Signaling Tech (Massachusetts, USA).

### GLP-1 secretion assay in vitro

STC-1 cells were seeded into six-well plates at a density of 2 × 10^5^ cells/well for 48 h; the cells were then incubated with L-DMEM (5.6 mmol/L glucose) containing 10% FBS. After 3 h, the medium was subsequently removed, and the cells were incubated with or without 3DG at final concentrations of 80, 300 and 1000 ng/mL in 0.2% BSA H-DMEM (25 mmol/L) containing 5 × 10^−7^ M insulin for 6 h. After the incubation, the medium was collected and centrifuged at 12,000×*g* for 5 min at 4 °C to remove any floating cells. GLP-1 concentration in the supernatant was measured by ELISA (Millipore, MA, USA).

### Statistical analysis

Results of the experimental studies are expressed as mean ± SD. Statistical significance of differences was analyzed by the Student’s t test or One-way analysis of variance. All p values ≤0.05 were considered statistically significant.

## Results

### Increased 3DG contents in intestinal tissues of rats 2 weeks after intragastric administration of 3DG

**Fig. 1:**
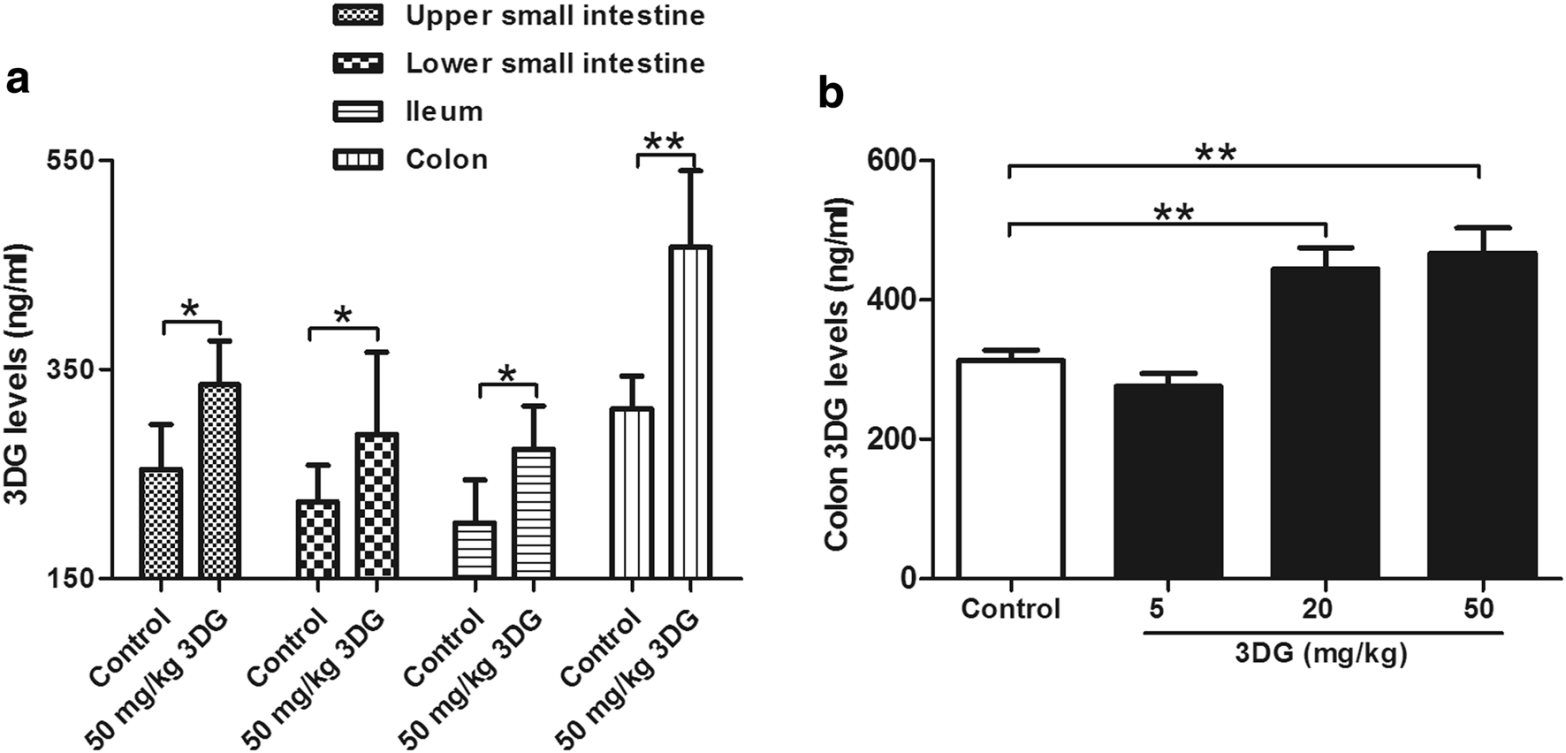
Increased 3DG contents in intestinal tissues of rats 2 weeks after intragastric administration of 3DG, *n* = 6 for each group. The upper small intestine, lower small intestine, ileum (**a**) and colon (**b**) 3DG levels were measured by HPLC after 2-week administration of 3DG or vehicle. Values are mean ± SD. *p < 0.05, **p < 0.01 compared with control group

Since lower absorption rate of 3DG has been indicated in in a single administration study [32], we further assess whether 3DG is capable of accumulating in intestinal tissue after continuous oral administration of 3DG. After intragastric administration of 50 mg/kg 3DG for 2 weeks, 3DG levels were increased significantly in the upper small intestine (1.4-fold), lower small intestine (1.4-fold), ileum (1.4-fold) and colon (twofold) compared with the basal levels in the corresponding control group. The colon had the greatest increase in the level of 3DG compared with control and had the highest levels among the tissue tested (Fig. 1a). Colon 3DG level was increased dependent on the concentration of 3DG administrated (Fig. 1b). A certain amount of 3DG in intestinal tissue of control rats may originate from intake of exogenous 3DG and production of 3DG in gut, which should be examined in a following study. These observations suggest that 3DG is capable of accumulating in intestinal tissue after long-term regularly intake of dietary 3DG.

### Intragastric administration of 3DG for 2 weeks led to a decrease in GLP-1 secretion in rats

**Fig. 2:**
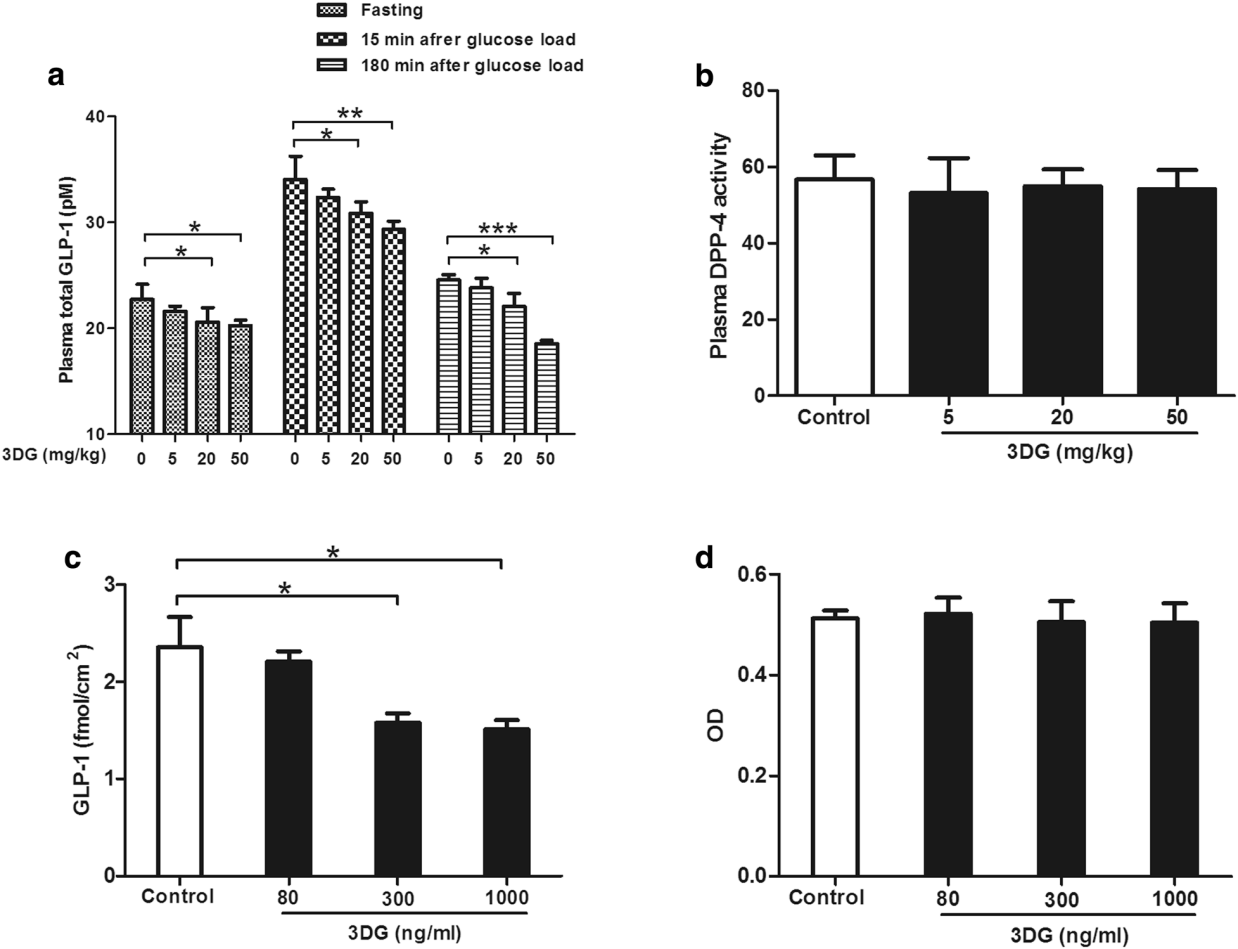
Intragastric administration of 3DG for 2 weeks led to a decrease in GLP-1 secretion in rats *n* = 6 for each group. After 2 weeks of intragastric administration of 3DG, an OGTT was performed in rats fasted overnight. Plasma total GLP-1 concentrations (**a**) were measured with ELISA kits before and after oral glucose load (2.5 g/kg) in 3DG (5, 20, 50 mg/kg) or vehicle treated rats. **b** Basal plasma DPP-4 activity was measured in 3DG (5, 20, 50 mg/kg) or vehicle treated rats. **c** STC-1 cells were exposed to different concentrations of 3DG (80, 300, 1000 ng/mL) for 6 h, and thereafter GLP-1 concentration in the supernatant was measured by ELISA. **d** STC-1 cells were treated with different concentrations of 3DG (80, 300, 1000 ng/mL) for 12 h and thereafter their viability was measured using an MTT assay. Values are mean ± SD. *p < 0.05, **p < 0.01 compared with control group at the same point (**a**). *p < 0.05, **p < 0.01, ***p < 0.001 compared with control group (**a**, **b**, **c**, **d**)

In consideration of the well-known relationship between increasing endogenous GLP-1 secretion and improved glucose tolerance, secretion of the gut hormone GLP-1 has been suggested to be impaired in T2DM and in conditions associated with hyperglycemia. We next determined whether 2-week intragastric administration of 3DG as an independent factor for the development of prediabetes affected GLP-1 secretion. Under fasting conditions, plasma GLP-1 concentrations were significantly decreased upon intragastric administration of either 20 or 50 mg/kg of 3DG (Fig. 2a, vehicle vs. 20 mg/kg 3DG: 22.698 ± 1.466 pM vs. 20.572 ± 1.395 pM, *p < 0.05, n = 6; vehicle vs. 50 mg/kg 3DG: 22.698 ± 1.466 pM vs. 20.233 ± 0.5219 pM, *p < 0.05, n = 6). Furthermore, plasma GLP-1 concentrations markedly increased after oral glucose loading in every group. Whereas glucose-induced increment in GLP-1 concentrations at 15 min point were significantly attenuated in 3DG-treated rats with either 20 mg/kg dose or 50 mg/kg does. (Figure 2a, vehicle vs. 20 mg/kg 3DG: 34.048 ± 2.198 pM vs. 30.858 ± 1.093 pM, ^#^ *p* < 0.05, n = 6; vehicle vs. 50 mg/kg 3DG: 34.048 ± 2.198 pM vs. 29.35 ± 0.7828 pM, ^#^ *p* < 0.01, n = 6). Similarly, the plasma GLP-1 concentrations were significantly lower in 3DG-treated rats with either 20 mg/kg dose or 50 mg/kg does than that in control rats 180 min after the glucose load. In addition, we examined the plasma DPP-4 activity to determine whether the decreased plasma GLP-1 levels associated with 3DG treatment were due to the potentiation of DPP-4 or not. We found no significant differences in plasma DPP-4 activity between 3DG-treated groups and vehicle-treated group (Fig. 2b), which indicates a decrease in GLP-1 secretion in 3DG-treated rats. We next determined whether 3DG directly affects GLP-1 secretion from the L-cells, mouse enteroendocrine STC-1 cells were exposed to 3DG at concentrations similar to those obtained from intestinal tissues contents in 3DG-treated rats. As shown in Fig. 2c, GLP-1 secretion in response to treatment with 300 or 1000 ng/mL 3DG in regular culture media was significantly reduced. Furthermore, under the conditions tested 3DG at concentrations of 80, 300 and 1000 ng/mL failed to alter STC-1 cell viability (Fig. 2d). These results indicated an impaired GLP-1 secretion in 3DG-treated rats.

### Reduced expressions of TAS1R2, TAS1R3 and TRPM5 in both duodenum and colon of rats 2 weeks after intragastric administration of 3DG

**Fig. 3:**
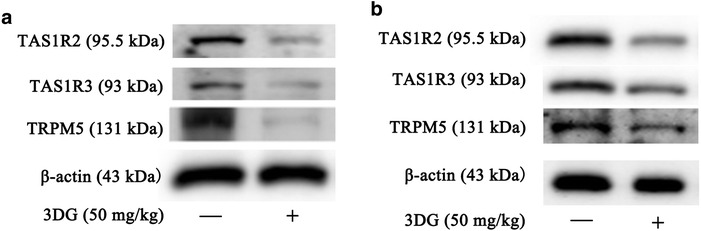
Reduced expressions of TAS1R2, TAS1R3 and TRPM5 in both duodenum and colon of rats 2 weeks after intragastric administration of 3DG *n* = 6 for each group. After 2 weeks of intragastric administration of 50 mg/kg 3DG, the duodenum and colon tissues were freshly isolated from rats. Representative western blotting analysis of protein expressions in duodenum (**a**) and colon (**b**) tissues using specific antibodies against TAS1R2, TAS1R3 and TRPM5

Since sweet taste receptors (TAS1R2-TAS1R3) in intestine have been demonstrated to regulate GLP-1 secretion following sugar ingestion, we further examine the expressions of sweet receptor subunits and its downstream molecular TRPM5 in duodenum and colon. After 50 mg/kg 3DG administrated to rats for 2 weeks by gastric gavage, the protein expressions of TAS1R2, TAS1R3 and TRPM5 in both duodenum (Fig. 3a) and colon (Fig. 3b) were significantly decreased. These observations further suggest a decreased GLP-1 secretion in 3DG-treated rats.

### Reduced plasma insulin concentrations and elevated plasma glucagon concentrations in rats 2 weeks after intragastric administration of 3DG

**Fig. 4:**
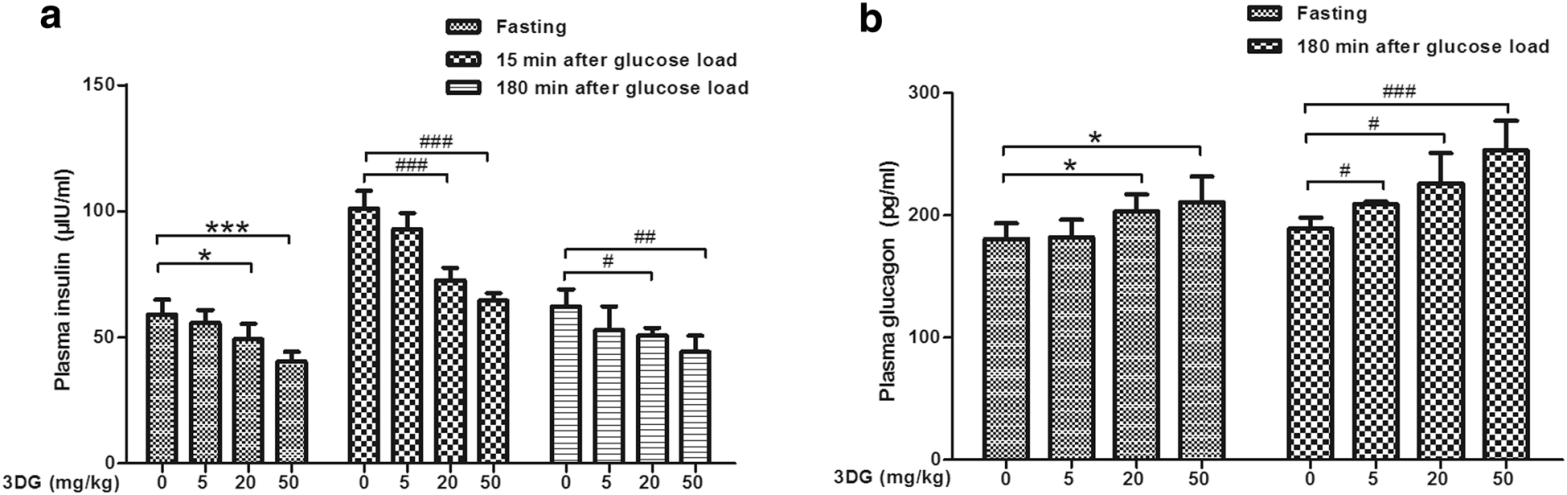
Reduced plasma insulin concentrations and elevated plasma glucagon concentrations in rats 2 weeks after intragastric administration of 3DG, *n* = 6 for each group. Plasma insulin concentrations (**a**) and glucagon concentrations (**b**) were measured before and after oral glucose load (2.5 g/kg) in 3DG (5, 20, 50 mg/kg) or vehicle-treated rats for 2 weeks. Values are mean ± SD. ^#^ *p* < 0.05, ^#^ *p* < 0.01 ^###^ *p* < 0.001 compared with control group at the same point (**a**, **b**). *p < 0.05, ***p < 0.001 compared with control group (**a**, **b**)

Reduced plasma GLP-1 levels sometimes observed in T2DM have been suggested to be associated with an impaired insulin secretion. We examined plasma insulin concentrations before and 15 and 180 min points after an oral glucose load in rats. Under fasting conditions, plasma insulin concentrations were significantly lower in 20 and 50 mg/kg 3DG-treated groups than that in control group (Fig. 4a). Plasma insulin concentrations markedly increased at 15 min in every group, whereas increase in plasma insulin concentrations was significantly lower in 3DG-treated rats with either 20 mg/kg dose or 50 mg/kg does than that in control rats (Fig. 4a). Plasma insulin concentrations at 180 min returned to the basal level in every group, and 20 and 50 mg/kg 3DG-treated rats displayed lower insulin concentrations than those in the control rats at 180 min (Fig. 4a). Additionally, plasma glucagon concentrations before and 180 min after oral glucose load in rats were also examined. As shown in Fig. 4b, plasma glucagon concentrations in 3DG-treated rats with either 20 mg/kg dose or 50 mg/kg does were higher than that in control rats 180 min after oral glucose loading. Similarly, the plasma glucagon concentrations at fasting conditions were significantly higher in 3DG-treated rats with either 20 mg/kg dose or 50 mg/kg does than that in control rats (Fig. 4b). These results indicated that 3DG-treated rats displayed obviously pancreatic islet cell dysfunction that is one of the typical characteristic of T2DM.

### Intragastric administration of 3DG for 2 weeks caused normal rats to develop elevated fasting blood glucose concentration and impaired oral glucose tolerance

**Fig. 5:**
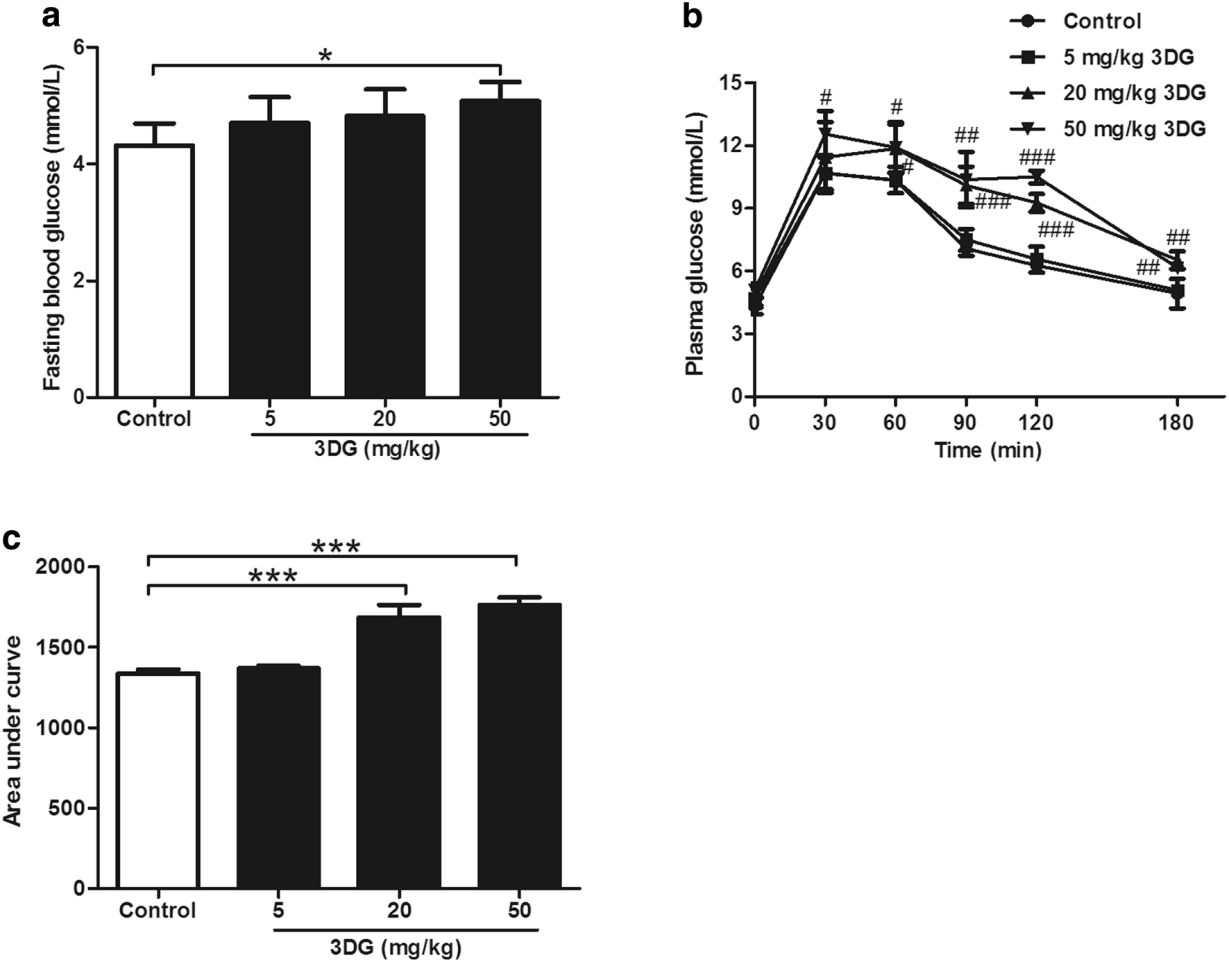
Intragastric administration of 3DG for 2 weeks caused normal rats to develop increased fasting blood glucose concentration and impaired oral glucose tolerance *n* = 6 for each group. **a** Fasting plasma glucose levels were measured in rats after 2 weeks of 3DG (5, 20, 50 mg/kg) or vehicle treated. **b** OGTT (2.5 g/kg) was performed after 2-week administration of 3DG (5, 20, 50 mg/kg) or vehicle in rats. **c** The glycaemic response was expressed as the area under the curve. Values are mean ± SD. ^#^ *p* < 0.05, ^##^ *p* < 0.01 ^###^ *p* < 0.001 compared with control group at the same point (**b**). *p < 0.05, ***p < 0.001 compared with control group (**a**, **c**)

3DG has been suggested as an independent factor for the development of prediabetes. We also evaluated the effect of 3DG treatment on fasting blood glucose and oral glucose tolerance in rats. After oral administration of 20 and 50 mg/kg 3DG, the fasting blood glucose level of 3DG-treated groups were significantly higher than that of vehicle-treated group (vehicle vs. 20 mg/kg 3DG: 4.32 ± 0.376 mmol/L vs. 4.89 ± 0.278 mmol/L, *p < 0.05; vehicle vs. 50 mg/kg 3DG: 4.32 ± 0.376 mmol/L vs. 5.08 ± 0.327 mmol/L, *p < 0.05) (Fig. 5a). As shown in Fig. 5b, the groups of 3DG-treated rats had impaired oral glucose tolerance in dose-dependent manner when compared to that of vehicle-treated group (vehicle vs. 20 mg/kg 3DG: at 30, 60, 90, 120, 180 min, ^#^ *p* < 0.05, ^#^ *p* < 0.05, ^##^ *p* < 0.01, ^###^ *p* < 0.001, ^##^ *p* < 0.01, respectively; vehicle vs. 50 mg/kg 3DG: at 30, 60, 90, 120, 180 min, ^#^ *p* < 0.05, ^#^ *p* < 0.05, ^##^ *p* < 0.01, ^###^ *p* < 0.001, ^##^ *p* < 0.01, respectively). Consistent results were obtained when the glycaemic response was expressed as the area under the curve (AUC) (Fig. 5c).

## Discussion

The objective of this study was to investigate whether 3DG is capable of accumulating in intestinal tissue of Sprague–Dawley (SD) rats after 2-week administration of 3DG by gastric gavage and if so, the effects of intragastric administration of 3DG on plasma levels of GLP-1, insulin and glucagon, and glucose regulation are further investigated. We demonstrated for the first time that intragastric administration of 3DG to rats for 2 weeks led to an obvious increase in 3DG content of the upper small intestine, lower small intestine, ileum and colon, and reduced plasma total GLP-1 and insulin concentrations in a similar manner, in conjunction with increased fasting blood glucose concentration and reduced oral glucose tolerance. The reduced plasma GLP-1 levels occurred in conjunction with reduced expressions of TAS1R2, TAS1R3 and TRPM5 in duodenum and colon and plasma dipeptidyl peptidase-4 activity was not altered, which suggested a reduced GLP-1 secretion in 3DG-treated rats. Moreover, non-cytotoxic concentrations of 3DG directly attenuated GLP-1 secretion in STC-1 cells. From this study, we also observed that 3DG-treated rats displayed obviously pancreatic islet cell dysfunction characterized by decreased plasma insulin level and elevated plasma glucagon level, associated with the development of impaired glucose regulation (IGR). Body weight was equivalent between 3DG-treated rats and vehicle-treated rats (data not shown).

A recent study on the influence of dietary on metabolism of 3DG in healthy volunteers experimented by Julia Degen et al. [34]., who speculated that orally ingested 3DG remained in the content of gastrointestinal tract to a major degree. As previous reports have indicated that the absorption rate of 3DG from foodstuffs is very slow in a single administration study [32]. This idea is further strengthened by the results that 3DG content of intestinal tissues was significantly higher in rats 2 weeks after intragastric administration of a high dosage (50 mg/kg) of 3DG than control rats, especially in colon section (Fig. 1a). Furthermore, increased 3DG content in colon section was also observed in rats after administration of a lower dosage (20 mg/kg) of 3DG (Fig. 1b). Similar with the distribution of 3DG in intestinal tissue, it has been reported that GLP-1 is secreted postprandially by intestinal L-cells that increase in density along the intestine and are found in highest amount in the colon [35]. In the present study, plasma GLP-1 concentrations decreased after intragastric administration of 3DG (Fig. 2a). Furthermore, at concentrations similar to those obtained from intestinal tissues contents in 3DG-treated rats, 3DG directly reduced GLP-1 secretion in the STC-1 cells in a dose-dependent manner (Fig. 2c) together with the unaltered plasma DPP-4 activity in 3DG-treated rats (Fig. 2b), indicating that accumulation of 3DG in intestinal tissue could reduce GLP-1 secretion in rats. Additional, although no effect was observed in response to 5 mg/kg of 3DG on any of the parameters, the 5 mg/kg 3DG group had similar GLP-1 content in colon section compared with the control group, and further support for the notion that decreased GLP-1 secretion was the result of increased 3DG content in intestinal tissues. This idea was also supported by the results that 3DG-treated rats displayed reduced expressions of TAS1R2, TAS1R3 and TRPM5 in duodenum and colon (Fig. 3). Several lines of evidence have demonstrated that sweet taste receptors in intestine regulate GLP-1 secretion following sugar ingestion [36, 37]. Furthermore, disruption of sweet taste receptors action in animal experiments and L-cell model, using antagonists or genetic manipulation, displayed significantly reduced glucose-stimulated GLP-1 secretion [36–38]. Therefore, the attenuated GLP-1 secretion in 3DG-treated rats could be responsible for the decreased plasma GLP-1 concentrations. In addition, there was no significant difference in AGEs levels in the colon section between 20 mg/kg 3DG-treated group and the corresponding control group (Additional file 1: Figure S1). These results clearly indicates that 3DG was capable of accumulating in intestinal tissue of rats 2 weeks after administration of 3DG, which led to reduced GLP-1 secretion independently from AGEs action. Additionally, reduced expressions of TAS1R2, TAS1R3 and TRPM5 in duodenum and colon (Fig. 3) also provide an explanation for the results that 3DG treated decreased GLP-1 secretion in vitro and in vivo. Research for the confirmation of this mechanism is in progress. Although treatment of STC-1 cells with 3DG at concentrations similar to those obtained from intestinal tissues contents in 3DG-treated rats failed to alter cell viability (Fig. 2d), whether intragastric administration of 3DG for 2 weeks also could result in increased apoptosis of GLP-1-secreting cells in vivo remains unknown and deserves to be further investigated.

IGR, sometimes referred to as prediabetes including isolated impaired glucose tolerance (IGT), isolated impaired fasting glucose (IFG) or combined IGT/IFG, is a high risk state for developing diabetes [39]. It has been reported that intragastric administration of 3DG for 2 weeks increased plasma glucose level under oral glucose tolerance tests (OGTT) in normal mice [15]. Such an effect was also observed in our present study. The intragastric administration of 3DG for 2 weeks caused normal SD rats to develop IFG (Fig. 5a) in conjunction with IGT (Fig. 5b) and increased AUC (Fig. 5c) in dose-dependent manner. Furthermore, treatment with 3DG resulted in reduction of GLP-1 secretion (Fig. 2) and sweet taste receptors expression in duodenum and colon (Fig. 3). In support of the observations by other studies, (i) disruption of the GLP-1 receptor action in mice caused IFG and IGT [40]; (ii) reduced glucose tolerance was observed in the TAS1R3^−/−^ mice [41]. Additionally, we also observed the elevated plasma glucagon levels in 3DG-treated in addition to decreased plasma insulin levels (Fig. 4). The main pathophysiological feature of T2DM is pancreatic islet cell dysfunction which manifests as both insufficient insulin secretion from β cells and inappropriately elevated glucagon secretion from α-cell [42]. Therefore, we obtained a conclusion that 3DG-treated SD rats displayed typical pancreatic islet cell dysfunction, suggesting the pancreatic islet cell dysfunction occurred prior to the development of T2DM.

It is now generally accepted that GLP-1 has a broad role in glucose homeostasis, in great part through stimulation of nutrient-induced insulin secretion from pancreatic β-cells [35]. In healthy individuals, insulinotropic effects of GLP-1 accounted for 50–70% of prandial insulin secretion from pancreatic β-cells [43]. As previous reports have documented that administration of GLP-1 to T2DM significantly enhanced and may even restore to normal glucose-induced insulin secretion [44, 45]. In T2DM, reduced postprandial GLP-1 concentrations in T2DM have been suggested to result in an impaired insulin secretion [23]. This hypothesis is supported by the results that intragastric administration of 3DG for 2 weeks decreased plasma GLP-1 concentrations at fasting and 15 and 180 min points during an oral glucose load in rats (Fig. 2a) and at the same points with insulin (Fig. 4a). In addition, disruption of GLP-1 action in animal experiments, using antagonists or genetic manipulation, displayed significantly reduced insulin secretion [46, 47]. From above, it was concluded that the decreased plasma GLP-1 concentrations in rats induced by intragastric administration of 3DG resulting from a decreased GLP-1 secretion led to reduced plasma insulin concentrations and thereby resulted in IGR. Thus, the reduced GLP-1 secretion sometimes observed may explain part of impaired incretin effect in T2DM. Additionally, (i) GLP-1 is known to induce the β-cells proliferation, and GLP-1R^−/−^ mice exhibit increased susceptibility to β-cell apoptosis injury [48]; (ii) GLP-1 also reduces glucagon secretion, and the GLP-1 secretion in present study was accompanied by an increased in plasma glucagon concentrations (Fig. 4). These observations also support the above suspection. Thus, a decrease in the biological function of GLP-1 from reduced GLP-1 secretion could result in an impaired insulin secretion but may not the only cause. For example, whether intragastric administration of 3DG for 2 weeks increases plasma 3DG levels in rats is unknown. And if so, whether increased plasma 3DG directly affects insulin secretion from β-cells will be investigated.

## Conclusions

Our study demonstrated for the first time that 3DG was capable of accumulating in intestinal tissue and decreased secretion of GLP-1 and insulin in a similar manner in rats after 2-week oral administration of 3DG. We also found that 3DG-treated rats displayed obviously pancreatic islet cell dysfunction that is one of the typical characteristic of T2DM. Our data indicate the possibility that a decrease in the biological function of GLP-1 resulting from the decreased GLP-1 secretion by the accumulation of intestinal tissue 3DG is the most primary mechanism for the impaired insulin secretion, which ultimately promoted the development of IGR. These results provide insights into a potential action pathway linking some non-digestible dietary ingredients intake and development of diabetes. It will also contribute to a better understanding of the significance for restoring physiological GLP-1 secretion, and could lead to a new strategy to prevent the development of prediabetes or reverse IGR states.

## Additional file

**Additional file 1: Figure S1.** AGEs levels in the colon section of Sprague–Dawley rats after 2-week administration of 3DG by gastric gavage, *n* = 6 for each group. AGEs contents of colon tissue were measured by HPLC after 2-week administration of 3DG or vehicle. Values are mean ± SD.

## Abbreviations

DPP-4: dipeptidyl peptidase-4
GLP-1: glucagon-like peptide-1
IGR: impaired glucose regulation
T2DM: type 2 diabetes mellitus
3DG: 3-deoxyglucosone
AGEs: advanced glycation end products

## References

[1] Parillo M, Riccardi G (2004). Diet composition and the risk of type 2 diabetes: epidemiological and clinical evidence. Br J Nutr.

[2] Hu FB, Van Dam RM, Liu S (2001). Diet and risk of type II diabetes: the role of types of fat and carbohydrate. Diabetologia.

[3] Popkin BM (2004). The nutrition transition: an overview of world patterns of change. Nutr Rev.

[4] Suez J, Korem T, Zeevi D, Zilberman-Schapira G, Thaiss CA, Maza O, Israeli D, Zmora N, Gilad S, Weinberger A, Kuperman Y, Harmelin A, Kolodkin-Gal I (2014). Artificial sweeteners induce glucose intolerance by altering the gut microbiota. Nature.

[5] Maessen DE, Hanssen NM, Scheijen JL, van der Kallen CJ, van Greevenbroek MM, Stehouwer CD (2015). Post-glucose load plasma α-dicarbonyl concentrations are increased in individuals with impaired glucose metabolism and type 2 diabetes: the CODAM study. Diabetes Care.

[6] Jiang G, Zhang L, Ji Q, Wang F, Xu H, Huang F (2002). Accumulation of plasma 3-deoxyglucosone impaired glucose regulation in Chinese seniors: implication for senile diabetes?. Diabetes Metab Syndr.

[7] Dhar A, Desai KM, Wu L (2010). Alagebrium attenuates acute methylglyoxal-induced glucose intolerance in Sprague–Dawley rats. Br J Pharmacol.

[8] Miele C, Riboulet A, Maitan MA, Oriente F, Romano C, Formisano P (2003). Human glycated albumin affects glucose metabolism in L6 skeletal muscle cells by impairing insulin-induced insulin receptor substrate (IRS) signaling through a protein kinase C alpha-mediated mechanism. J Biol Chem.

[9] Vlassara H, Striker GE (2011). AGE restriction in diabetes mellitus: a paradigm shift. Nat Rev Endocrinol.

[10] Degen J, Hellwig M, Henle T (2012). 1,2-Dicarbonyl compounds in commonly consumed foods. J Agric Food Chem.

[11] Niwa T (1999). 3-Deoxyglucosone: metabolism, analysis, biological activity, and clinical implication. J Chromatogr B Biomed Sci Appl.

[12] Sakiyama H, Takahashi M, Yamamoto T, Teshima T, Lee SH, Miyamoto Y (2006). The internalization and metabolism of 3-deoxyglucosone in human umbilical vein endothelial cells. J Biochem.

[13] Liang G, Wang F, Song X, Zhang L, Qian Z, Jiang G (2016). 3-Deoxyglucosone induces insulin resistance by impairing insulin signaling in HepG2 cells. Mol Med Rep.

[14] Liang G, Song X, Xu H, Wang F, Zhang L, Zhou L (2016). 3-Deoxyglucosone induced acute glucose intolerance in Sprague–Dawley rats: involvement of insulin resistance and impaired β-cell function. Exp Clin Endocrinol Diabetes.

[15] Wang Q, Gr J (2010). Effects of 3-deoxyglucosone on blood glucose of normal mice. Chin J Diabetes.

[16] Unger RH, Eisentraut AM (1969). Entero-insular axis. Arch Intern Med.

[17] Miyawaki K, Yamada Y, Yano H, Niwa H, Ban N (1999). Glucose intolerance caused by a defect in the entero-insular axis: a study in gastric inhibitory polypeptide receptor knockout mice. Proc Natl Acad Sci USA.

[18] Meier JJ (2009). The contribution of incretin hormones to the pathogenesis of type 2 diabetes. Best Pract Res Clin Endocrinol Metab.

[19] Ørskov C (1992). Glucagon-like peptide-1, a new hormone of the entero-insular axis. Diabetologia.

[20] Sandoval DA, D’Alessio DA (2015). Physiology of proglucagon peptides: role of glucagon and GLP-1 in health and disease. Physiol Rev.

[21] Hira T, Ikee A, Kishimoto Y, Kanahori S, Hara H (2015). Resistant maltodextrin promotes fasting glucagon-like peptide-1 secretion and production together with glucose tolerance in rats. Br J Nutr.

[22] Cani PD, Daubioul CA, Reusens B, Remacle C, Catillon G, Delzenne NM (2005). Involvement of endogenous glucagon-like peptide-1(7-36) amide on glycaemia-lowering effect of oligofructose in streptozotocin-treated rats. J Endocrinol.

[23] Vilsbøll T, Krarup T, Deacon CF, Madsbad S, Holst JJ (2001). Reduced postprandial concentrations of intact biologically active glucagon-like peptide 1 in type 2 diabetic patients. Diabetes.

[24] Yabe D, Kuroe A, Lee S, Watanabe K, Hyo T, Hishizawa M (2010). Little enhancement of meal-induced glucagon-like peptide 1 secretion in Japanese: comparison of type 2 diabetes patients and healthy controls. J Diabetes Investig.

[25] Legakis IN, Tzioras C, Phenekos C (2003). Decreased glucagon-like peptide 1 fasting levels in type 2 diabetes. Diabetes Care.

[26] Zhang F, Tang X, Cao H, Lü Q, Li N (2012). Impaired secretion of total glucagon-like peptide-1 in people with impaired fasting glucose combined impaired glucose tolerance. Int J Med Sci.

[27] Nauck M, Stöckmann F, Ebert R, Creutzfeldt W (1986). Reduced incretin effect in type 2 (non-insulin-dependent) diabetes. Diabetologia.

[28] Lugari R, Dei Cas A, Ugolotti D, Finardi L, Barilli AL, Ognibene C (2002). Evidence for early impairment of glucagon-like peptide 1-induced insulin secretion in human type 2 (non insulin-dependent) diabetes. Horm Metab Res..

[29] Cani PD, Holst JJ, Drucker DJ, Delzenne NM, Thorens B, Burcelin R (2007). GLUT2 and the incretin receptors are involved in glucose-induced incretin secretion. Mol Cell Endocrinol.

[30] Kappe C, Zhang Q, Nyström T, Sjöholm A (2014). Effects of high-fat diet and the anti-diabetic drug metformin on circulating GLP-1 and the relative number of intestinal L-cells. Diabetol Metab Syndr.

[31] Lei L, Wang J, Zhang Z, Zhang H, Chen H, Cai D (2013). Lipopolysaccharide-induced apoptosis in a murine intestinal endocrine cell line by modulation of Bcl-2, Baxand caspase-3. Mol Med Rep.

[32] Kato H, van Chuyen N, Shinoda T, Sekiya F, Hayase F (1990). Metabolism of 3-deoxyglucosone, an intermediate compound in the Maillard reaction, administered orally or intravenously to rats. Biochim Biophys Acta.

[33] Pederson RA, White HA, Schlenzig D, Pauly RP, McIntosh CH, Demuth HU (1998). Improved glucose tolerance in Zucker fatty rats by oral administration of the dipeptidyl peptidase IV inhibitor isoleucine thiazolidide. Diabetes.

[34] Degen J, Beyer H, Heymann B, Hellwig M, Henle T (2014). Dietary influence on urinary excretion of 3-deoxyglucosone and its metabolite 3-deoxyfructose. J Agric Food Chem.

[35] Baggio LL, Drucker DJ (2007). Biology of incretins: GLP-1 and GIP. Gastroenterology.

[36] Jang HJ, Kokrashvili Z, Theodorakis MJ, Carlson OD, Kim BJ, Zhou J (2007). Gut-expressed gustducin and taste receptors regulate secretion of glucagon-like peptide-1. Proc Natl Acad Sci USA.

[37] Margolskee RF, Dyer J, Kokrashvili Z, Salmon KS, Ilegems E, Daly K (2007). T1R3 and gustducin in gut sense sugars to regulate expression of Na+-glucose cotransporter 1. Proc Natl Acad Sci USA.

[38] Geraedts MC, Takahashi T, Vigues S, Markwardt ML, Nkobena A, Cockerham RE (2012). Transformation of postingestive glucose responses after deletion of sweet taste receptor subunits or gastric bypass surgery. Am J Physiol Endocrinol Metab.

[39] Tabák AG, Herder C, Rathmann W, Brunner EJ, Kivimäki M (2012). Prediabetes: a high-risk state for diabetes development. Lancet.

[40] Scrocchi LA, Marshall BA, Cook SM, Brubaker PL, Drucker DJ (1998). Identification of glucagon-like peptide 1 (GLP-1) actions essential for glucose homeostasis in mice with disruption of GLP-1 receptor signaling. Diabetes.

[41] Murovets VO, Bachmanov AA, Travnikov SV, Churikova AA, Zolotarev VA (2014). The involvement of the T1R3 receptor protein in the control of glucose metabolism in mice at different levels of glycemia. J Evol Biochem Physiol..

[42] Göke B (2008). Islet cell function: alpha and beta cells–partners towards normoglycaemia. Int J Clin Pract.

[43] Meier JJ, Nauck MA (2005). Glucagon-like peptide 1 (GLP-1) in biology and pathology. Diabetes Metab Res Rev.

[44] Kjems LL, Volund A, Madsbad S (1999). The effect of exogenous GLP-1 on the glucose mediated insulin secretion: a dose-response study in patients with type 2 diabetes mellitus and control subjects. Diabetologia.

[45] Nauck MA, Heimesaat MM, Orskov C, Holst JJ, Ebert R, Creutzfeldt W (1993). Preserved incretin activity of glucagon-like peptide 1 [7-36 amide] but not of synthetic human gastric inhibitory polypeptide in patients with type-2 diabetes mellitus. J Clin Investig.

[46] Hansotia T, Drucker DJ (2005). GIP and GLP-1 as incretin hormones: lessons from single and double incretin receptor knockout mice. Regul Pept.

[47] Campbell JE, Drucker DJ (2013). Pharmacology, physiology, and mechanisms of incretin hormone action. Cell Metab.

[48] Li Y, Hansotia T, Yusta B, Ris F, Halban PA, Drucker DJ (2003). Glucagon-like peptide-1 receptor signaling modulates beta cell apoptosis. J Biol Chem.

