# Supplementary material for: Accumulation of intestinal tissue 3-deoxyglucosone attenuated GLP-1 secretion and its insulinotropic effect in rats
Source: Diabetol Metab Syndr. 2016 Nov 29;8:78. doi: 10.1186/s13098-016-0194-9 (PMC5129672; doi:10.1186/s13098-016-0194-9)
Supplement: Supplementary file 1 — Additional file 1: Figure S1. AGEs levels in the colon section of Sprague–Dawley rats after 2-week administration of 3DG by gastric gavage, n = 6 for each group. AGEs contents of colon tissue were measured by HPLC after 2-week administration of 3DG or vehicle. Values are mean ± SD. [file 13098_2016_194_MOESM1_ESM.doc]

**Supplement materials**


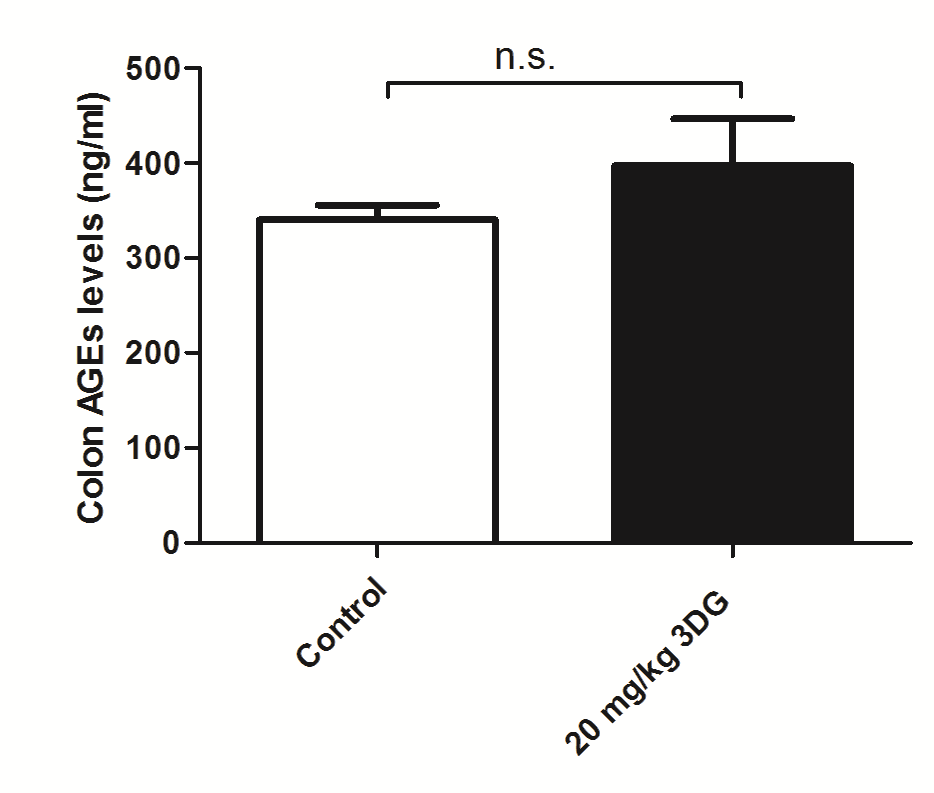


**EMS Figure 1 AGEs levels in the colon section of Sprague-Dawley rats after two-week administration of 3DG by gastric gavage.** *n*=6 for each group. AGEs contents of colon tissue were measured by HPLC after two-week administration of 3DG or vehicle. Values are mean±SD.
